# Supplementary material for: Selective HDAC6 inhibitor TubA offers neuroprotection after intracerebral hemorrhage via inhibiting neuronal apoptosis
Source: PeerJ. 2023 Apr 28;11:e15293. doi: 10.7717/peerj.15293 (PMC10150719; doi:10.7717/peerj.15293)
Supplement: Supplemental Information 1 — Garcia, Garcia test; WB, western blot; IF, immunofluorescence staining; BWC, brain water content; TUNEL, TdT-mediated dUTP-biotin nick end labeling staining; HE, Hematoxylin and eosin staining. [file peerj-11-15293-s001.docx]

**Supplementary Material 1**

**Table 1 Rats Assignment and Use**

| **Group** | **Garcia** | **WB** | **IF** | **BWC** | **TUNEL** | **HE** | **Rats**  **Used** | **Rats**  **Died** | **Rats**  **in Total** | **Mortality**  **Rate** |
| --- | --- | --- | --- | --- | --- | --- | --- | --- | --- | --- |
| **Experiment 1.**  **Time-course** |  |  |  |  |  |  |  |  |  |  |
| **Sham 4h** |  | 3 |  |  |  |  | **3** | 0 | **3** | 0% |
| **Sham 6h** |  | 3 |  |  |  |  | **3** | 0 | **3** | 0% |
| **Sham 12h** |  | 3 |  |  |  |  | **3** | 0 | **3** | 0% |
| **Sham 1d** |  | 3 |  |  |  |  | **3** | 0 | **3** | 0% |
| **Sham 3d** |  | 3 |  |  |  |  | **3** | 0 | **3** | 0% |
| **NO. of Sham** |  | **15** |  |  |  |  | **15** | **0** | **15** |  |
|  |  |  |  |  |  |  |  |  |  |  |
| **ICH 4h** |  | 3 |  |  |  |  | **3** | 0 | **3** | 0% |
| **ICH 6h** |  | 3 |  |  |  |  | **3** | 0 | **3** | 0% |
| **ICH 12h** |  | 3 |  |  |  |  | **3** | 1 | **4** | 25% |
| **ICH 1d** |  | 3 |  |  |  |  | **3** | 2 | **5** | 40% |
| **ICH 3d** |  | 3 |  |  |  |  | **3** | 1 | **4** | 25% |
| **NO. of ICH** |  | **15** |  |  |  |  | **15** | **4** | **19** |  |
|  |  |  |  |  |  |  |  |  |  |  |
| **Experiment 2.**  **Effect of TubA** |  |  |  |  |  |  |  |  |  |  |
| **Sham** | 10 | 3 |  | 10 |  |  | **23** | 0 | **23** | 0% |
| **ICH+Vehicle** | 10 | 3 |  | 10 |  |  | **23** | 11 | **34** | 32.4% |
| **ICH****+TubA 25mg/kg** | 10 | 3 |  | 10 |  |  | **23** | 4 | **27** | 14.8% |
| **ICH+TubA 40mg/kg** | 10 | 3 |  | 10 |  |  | **23** | 3 | **26** | 11.5% |
| **NO. of Rats** | **40** | **12** |  | **40** |  |  | **92** | **15** | **107** |  |
|  |  |  |  |  |  |  |  |  |  |  |
| **Experiment 3.**  **Mechanism** |  |  |  |  |  |  |  |  |  |  |
| **Sham^(1)^** |  | 3 | 3 |  | 3 | 3 | **12** | 0 | **12** | 0% |
| **ICH+Vehicle ^(2)^** |  | 3 | 3 |  | 3 | 3 | **12** | 6 | **18** | 33.3% |
| **ICH+TubA 25mg/kg** |  | 3 | 3 |  | 3 | 3 | **12** | 2 | **14** | 14.3% |
| **ICH+TubA 40mg/kg** |  | 3 | 3 |  | 3 | 3 | **12** | 1 | **13** | 7.7% |
| **NO. of Rats** |  | **12** | **12** |  | **12** | **12** | **48** | **7** | **55** |  |

**Garcia: Garcia test WB: western blot IF: immunofluorescence staining BWC: brain water content TUNEL: TdT-mediated dUTP-biotin nick end labeling staining HE: Hematoxylin and eosin staining**
