# Supplementary material for: Selective HDAC6 inhibitor TubA offers neuroprotection after intracerebral hemorrhage via inhibiting neuronal apoptosis
Source: PeerJ. 2023 Apr 28;11:e15293. doi: 10.7717/peerj.15293 (PMC10150719; doi:10.7717/peerj.15293)
Supplement: Supplemental Information 2 [file peerj-11-15293-s002.docx]

**Supplementary Material 2**

**Table 2 HDAC6 siRNA** **sequences.**

| **HDAC6 siRNA target** | **Sequence** |
| --- | --- |
| **target 1** | **CTTCGAAGCGAAATATTAAAA** |
| **target 2** | **GCAGTTAAATGAATTCCATTG** |
| **target 3** | **GAAACAACCCAGTACATGAAT** |

**The interference efficiency of three different siRNAs was tested, and we used target 3 in this study.**

**
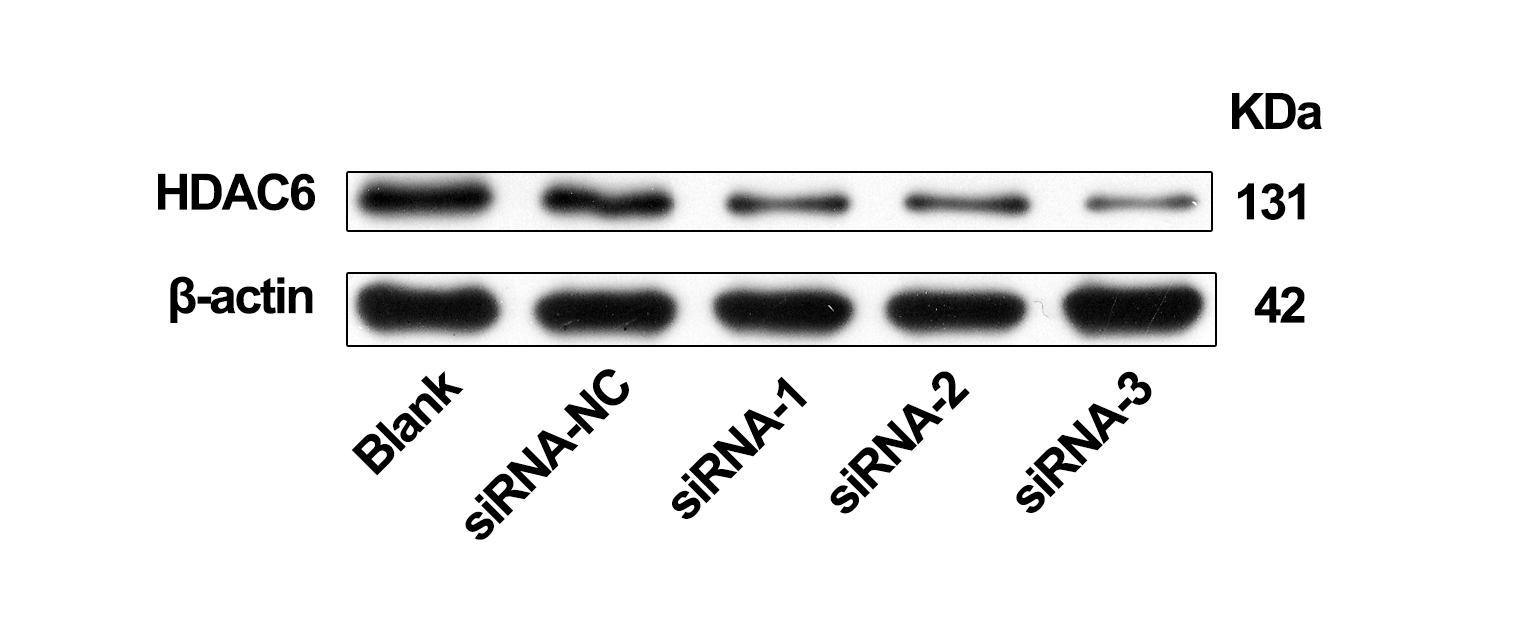
**
