# Supplementary figures and images for: Selective HDAC6 inhibitor TubA offers neuroprotection after intracerebral hemorrhage via inhibiting neuronal apoptosis
Source: PeerJ. 2023 Apr 28;11:e15293. doi: 10.7717/peerj.15293 (PMC10150719; doi:10.7717/peerj.15293)

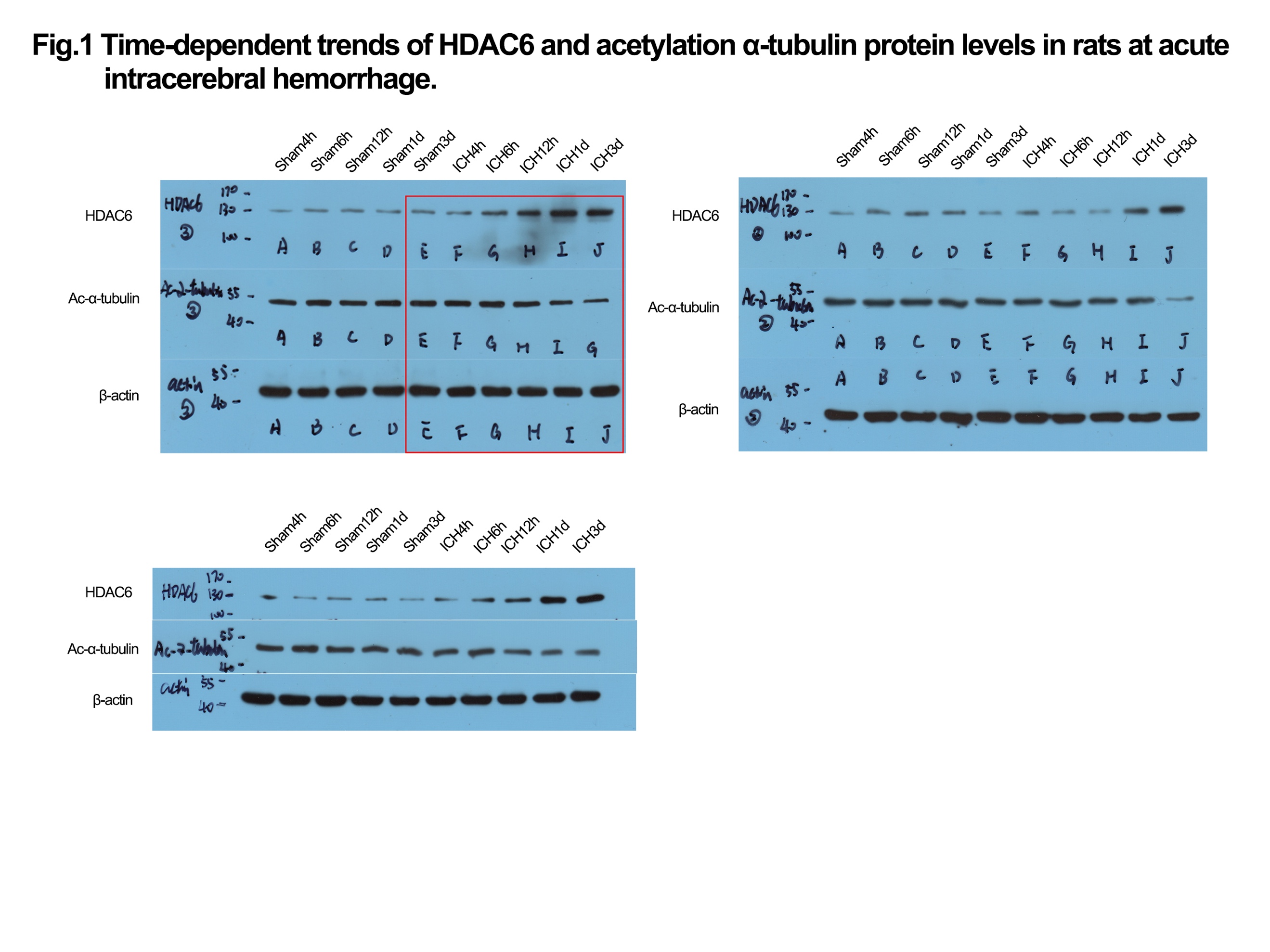


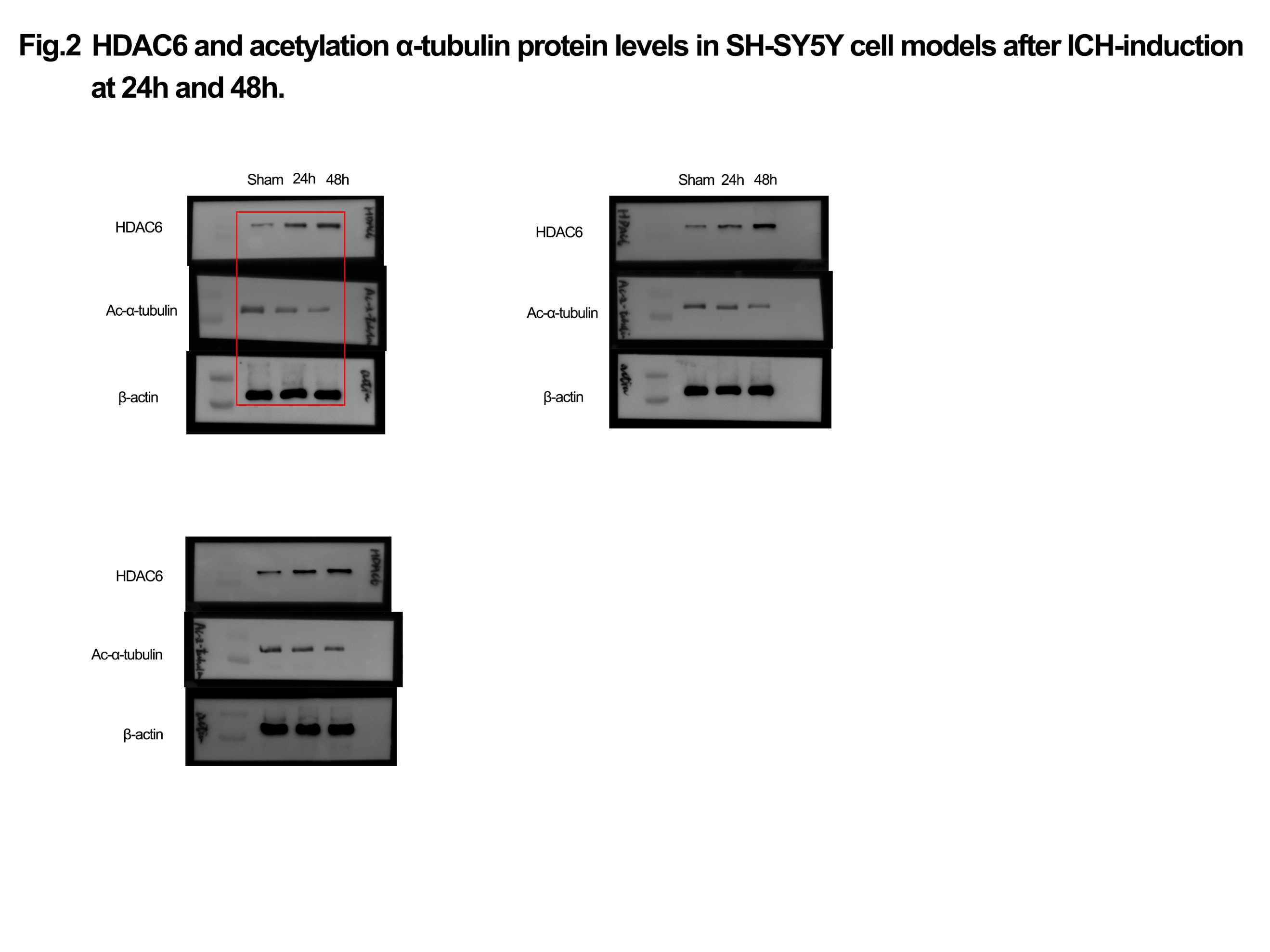


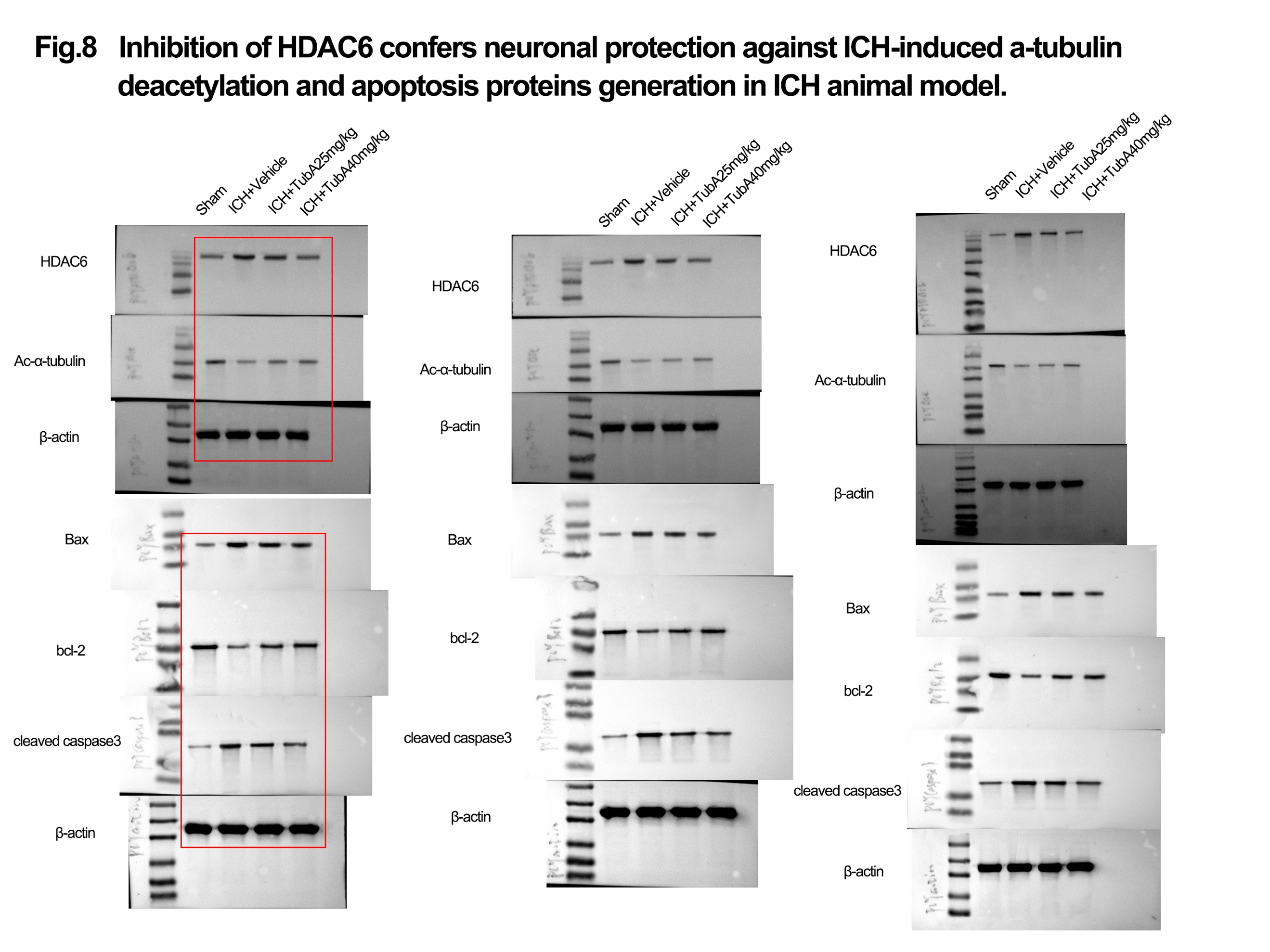


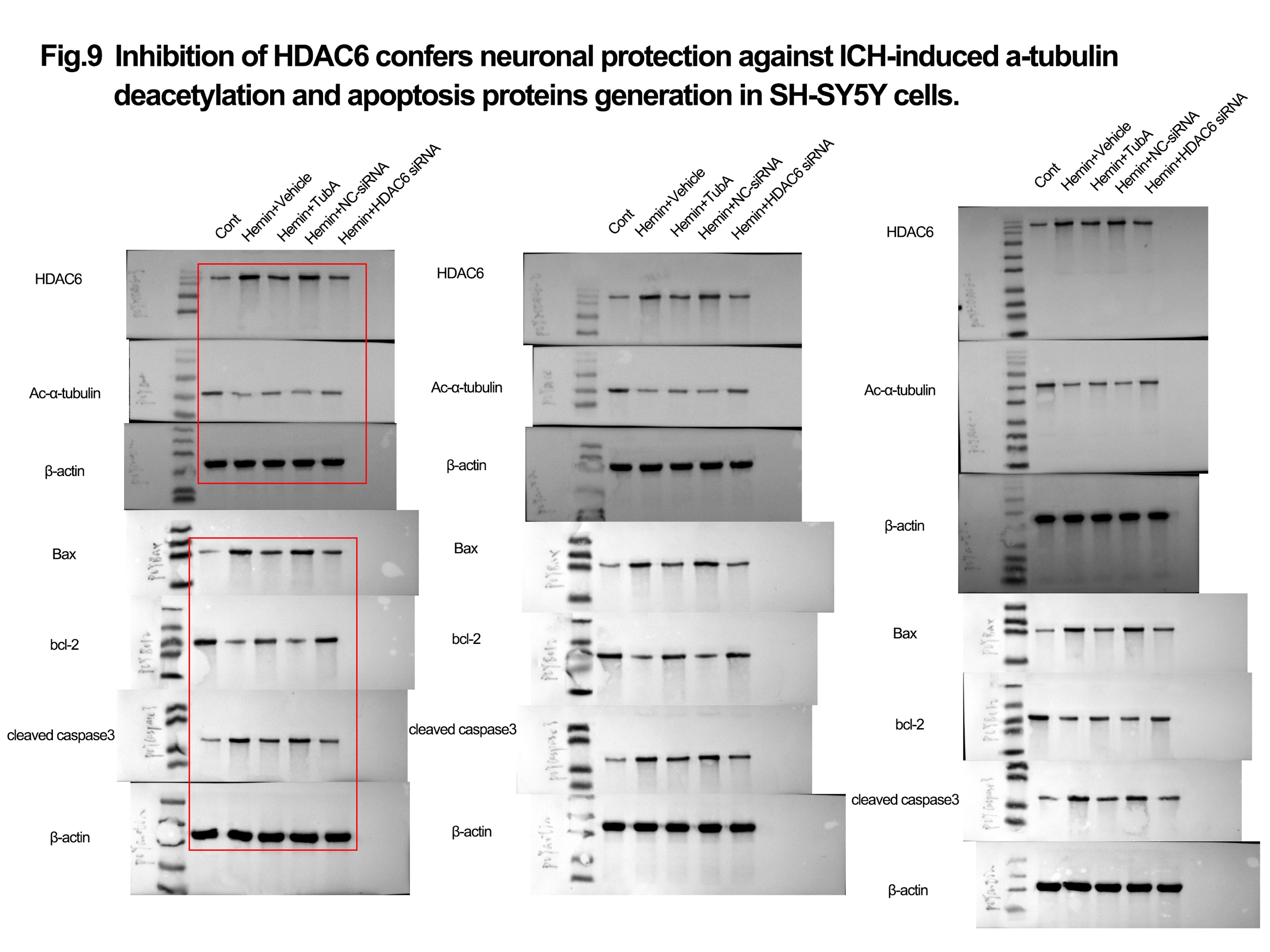

Supplement: Supplemental Information 3 [file peerj-11-15293-s003.docx]
